# The Evolution of Fluorescence-Guided Surgery

**DOI:** 10.1007/s11307-022-01772-8

**Published:** 2022-09-19

**Authors:** Stan Van Keulen, Marisa Hom, Haley White, Eben L. Rosenthal, Fred M Baik

**Affiliations:** 1 Department of Oral and Maxillofacial Surgery and Oral Pathology, Amsterdam UMC, Amsterdam, The Netherlands; 2 Department of Otolaryngology–Head and Neck Surgery, Vanderbilt University Medical Center, Nashville, TN USA; 3 Department of Otolaryngology–Head and Neck Surgery, Stanford University School of Medicine, Stanford, CA USA

**Keywords:** Fluorescence-guided surgery, Molecular imaging, Oncology

## Abstract

There has been continual development of fluorescent agents, imaging systems, and their applications over the past several decades. With the recent FDA approvals of 5-aminolevulinic acid, hexaminolevulinate, and pafolacianine, much of the potential that fluorescence offers for image-guided oncologic surgery is now being actualized. In this article, we review the evolution of fluorescence-guided surgery, highlight the milestones which have contributed to successful clinical translation, and examine the future of targeted fluorescence imaging.

## Introduction

Surgery remains the foundation for curative treatment in most solid tumors. Overall survival strongly correlates with the presence of residual cancer cells after resection, known as positive tumor margins. These positive margins are associated with increased local recurrence and poor prognosis in numerous cancers such as head and neck, brain, breast, lung, prostate, and gastrointestinal cancer. Today surgeons rely on visual and tactile cues to delineate cancer tissue from healthy adjacent tissue. Despite the use of frozen section analysis (i.e., rapid intraoperative histopathological assessment), overall positive margin rates across all cancer types have stagnated between 15 and 60% over the past few decades [1–4], indicating the need for more sufficient intraoperative tumor identification.

**Fig. 1:**
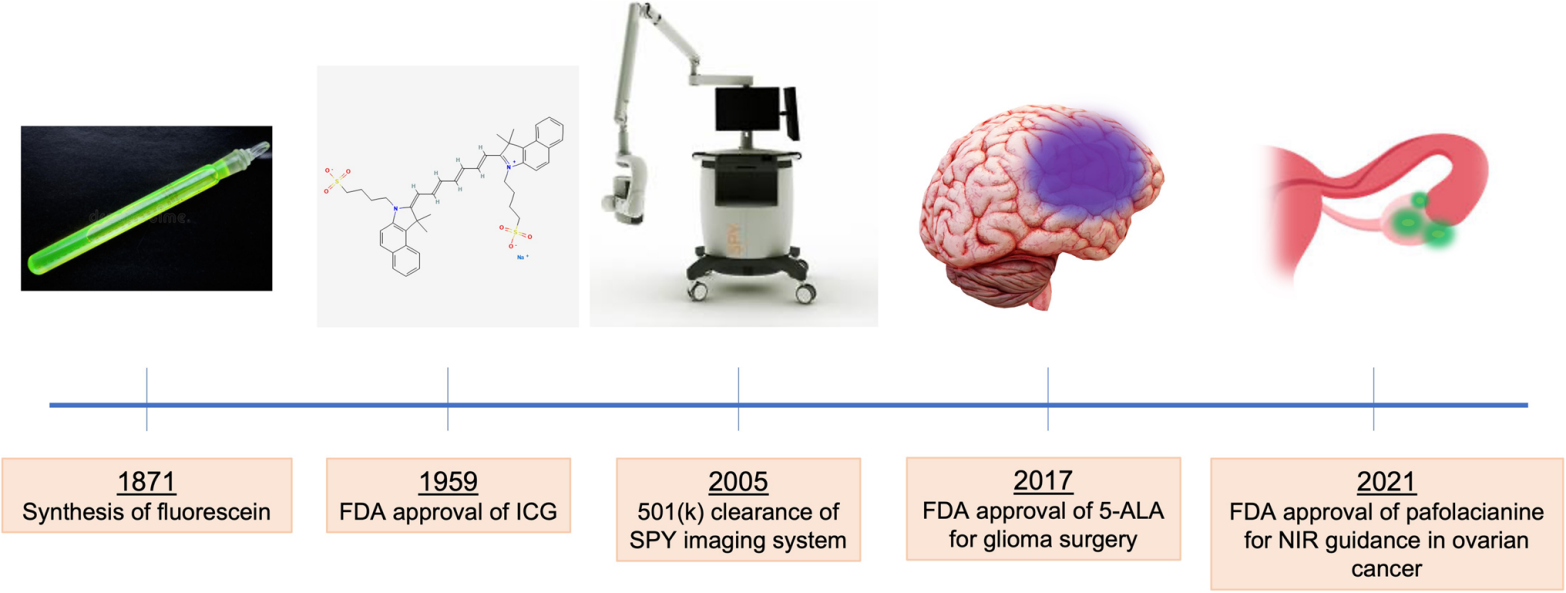
Milestones in the evolution of fluorescence-guided surgery. Fluorescein was initially discovered in the middle of the nineteenth century, but ICG became the first FDA-approved fluorescent dye. Subsequent clearance of the Novodaq SPY imaging system paved the way for device development, and a growing clinical experience has provided the foundation for FDA approval of fluorescent agents for intraoperative use.

Fluorescence has emerged as a compelling strategy to enhance surgical vision by highlighting tissue which may otherwise be indistinct from its surroundings. Many of the features unique to fluorescence has led to its widespread use in science and medicine. The ability of a fluorophore to absorb and emit light energy allows it to be easily recognized as compared to reflected white-light images. The translation of fluorescence in medicine and surgery has since exponentially grown, and its applications in surgery hold potential to dramatically impact current practice, the beginnings of which we are now witnessing with the recent FDA approvals of 5-aminolevulinic acid (5-ALA) for brain cancer [5], hexaminolevulinate for bladder cancer [6], and pafolacianine for ovarian cancer surgery [7, 8]. This article will review the beginnings of fluorescence-guided surgery (FGS) and describe key milestones which have facilitated the expansion of FGS into successful clinical translation (Fig. 1).

## Early Fluorescent Contrast Agents

While increasing numbers of agents are being developed for FGS (Table 1), four fluorescent dyes (fluorescein, methylene blue, 5-ALA, and indocyanine green) were developed or discovered more than seven decades ago, and their translation into clinical medicine have contributed to our growing experience in FGS.

**Table 1:** List of common fluorescent agents used in FGS

Fluorescence agent	Excitation (nm)	Emission (nm)	Route of administration	Administration-to-imaging time
Indocyanine green	807	822	Intravenous Intradermal	Minutes
Methylene blue	670	690	Intravenous Intradermal	Minutes
5-Aminolevulinic acid & derivatives	380–440	620	Oral Topical	2–4 h
Fluorescein sodium	465–490	520–530	Intravenous Topical	During induction
Folate (folate-FITC)	495	520	Intravenous	2–8 h
IRDye800DX conjugate	775	796	Intravenous	Various
IRDye700DX conjugate	680	687	Intravenous	Various
Pafolacianine (OTL38)	760–785	796	Intravenous	1–9 h

### Fluorescein

Fluorescein was first synthesized in 1871 by Adolf von Baeyer [9]. Fluorescein was immediately noted for its bright fluorescence, hence its given name, and found widespread value in many industries, including as a chemical stain (fluorescein reacts with bromine to produce eosin). It found use in medicine primarily as a diagnostic agent in ophthalmology and eventually obtained FDA approval for these indications in 1972, nearly a century after its discovery. Administered topically, fluorescein provides contrast to assist with routine ophthalmic exams, diagnosis of corneal lesions, and imaging of retinal vessels [10]. Fluorescein administered intravenously has shown usefulness in retinal and choroidal angiography [11]. Fluorescein is excited by cobalt blue light (465–490 nm) and has a peak emission between 520 and 530 nm. Because this emission peak is similar to that of hemoglobin, the application of fluorescein to indications outside of ophthalmology has largely been limited.

### Methylene Blue

Methylene blue was discovered in 1876 by Heinrich Caro, a colleague of von Baeyer [12]. Although the dye received FDA approval for its therapeutic use in treating methemoglobinemia, it is widely used off-label as an intradermally injected visual tracer for sentinel lymph node mapping [13]. While many studies have explored the use of intravenous methylene blue in tumor identification and delineation of urethral anatomy [14, 15], the potential for clinical translation is limited. Its fluorescence properties for use in FGS are not optimal, given its weak fluorescence and absorption/emission spectrum that lies within the visible light spectrum (excitation 670 nm, emission 690 nm).

### 5-ALA and Its Derivatives

5-ALA (5-aminolevulinic acid), a naturally occurring fluorescence precursor in the heme synthesis pathway, was discovered by David Shemin in the late 1940s [16]. 5-ALA is intracellularly metabolized to produce protoporphyrin IX, which fluoresces in the visible light spectrum (excitation 380–440 nm, emission 620 nm). Administered orally, 5-ALA has been shown to accumulate protoporphyrin IX in high-grade gliomas, allowing for fluorescence identification of tumors intraoperatively [17]. In addition, topical administration of hexaminolevulinate (the hexyl ester of 5-ALA) combined with blue light cystoscopy has demonstrated improved detection and decreased recurrence of non-muscle invasive bladder cancer (NMIBC) [6]. Stenzl et al., in a large, multinational, randomized control trial of 814 patients, showed that compared to white-light cystoscopy alone, the use of blue light cystoscopy with hexaminolevulinate reduced recurrence rates of NMIBC in a 9-month observation period (56% versus 47%, respectively, *p* = 0.026) [18]. Many other clinical trials have corroborated the value of these agents as intraoperative guides to improve disease outcomes, which have led to their eventual FDA approval [5, 19].

### Indocyanine Green

Indocyanine green (ICG) was initially developed in 1950s as a cyan dye for use in the film industry, as the introduction of color into traditional black and white film was occurring. The first description of ICG and its potential use in medicine is attributed to Irwin J. Fox and Earl H. Wood from the Mayo Clinic, who described to the use of indicator-dilution curves to assess cardiac output [20]. Their study led to FDA approval of the agent in 1959 for use in cardiac-output monitoring, leading to the marketing of ICG as “Cardio-green.” The success of ICG opened the doors to investigation of ICG in many other applications, including assessment of hepatic function [21] and ophthalmologic angiography [22].

In many ways, ICG represents an ideal fluorescent agent for use in intraoperative surgical guidance (Table 2). The safety, ease and swiftness of detection, and near-infrared (NIR) imaging capability of ICG have contributed to the widespread adoption [23]. Equally important to this success has been the development and approval of compatible imaging systems.

**Table 2:** Ideal properties of ICG useful for intraoperative surgical guidance

Property	ICG features	Significance
Fluorescence	• Excitation (807 nm) and emission (822 nm) lie in the near-infrared spectrum	Near-infrared imaging allows for increased depth of signal penetration and decreased autofluorescence from background tissue. Other modalities such as second-window and short-wave infrared imaging also apply
Route of administration	• Intravenous (0.1–0.5 mg/kg) • Intradermal	Rapid circulates through the vasculature allows for near real-time imaging. Intradermal route allows for lymphatic mapping
Pharmacokinetics	• Water-soluble, hydrophobic molecule, binds to plasma proteins • Short half-life (2–4 min)	ICG circulates and remains within the lymphovascular system and is rapidly cleared by the liver. This contributes to its excellent safety profile

### FDA Clearance of ICG Imaging Systems

Arguably, the most significant milestone in this regard was the 501(k) clearance of the SPY Imaging System in 2005. Developed by Novodaq Industries beginning in 1998, the intent of the original SPY system was to assess vascular perfusion using systemically administered ICG, to improve upon predicate devices which relied on X-ray or scanning laser alone for angiography [24]. Importantly, the clearance of the SPY system ushered a subsequent generation of intraoperative fluorescence imaging devices which utilized the SPY system as its predicate. This expansion of hardware development and subsequent 501(k) clearances have been crucial to supporting the parallel development of fluorescence agents. While the original SPY system has evolved to incorporate other indications, such as tissue perfusion in reconstructive surgery and gastrointestinal imaging (SPY SP2000, SP2001, SPY intraoperative imaging system), other devices have expanded on its capabilities, such as fluorescence overlay on reflected light (FLARE), ergonomics (PDE NEO, Artemis/Quest), multichannel functionality (Quest), and incorporation into microscopic, endoscopic, and robotic approaches (Firefly, Pinpoint, Leica/Zeiss) [25].

## Growing Clinical Applications for ICG and FGS

### Angiography

**Fig. 2:**
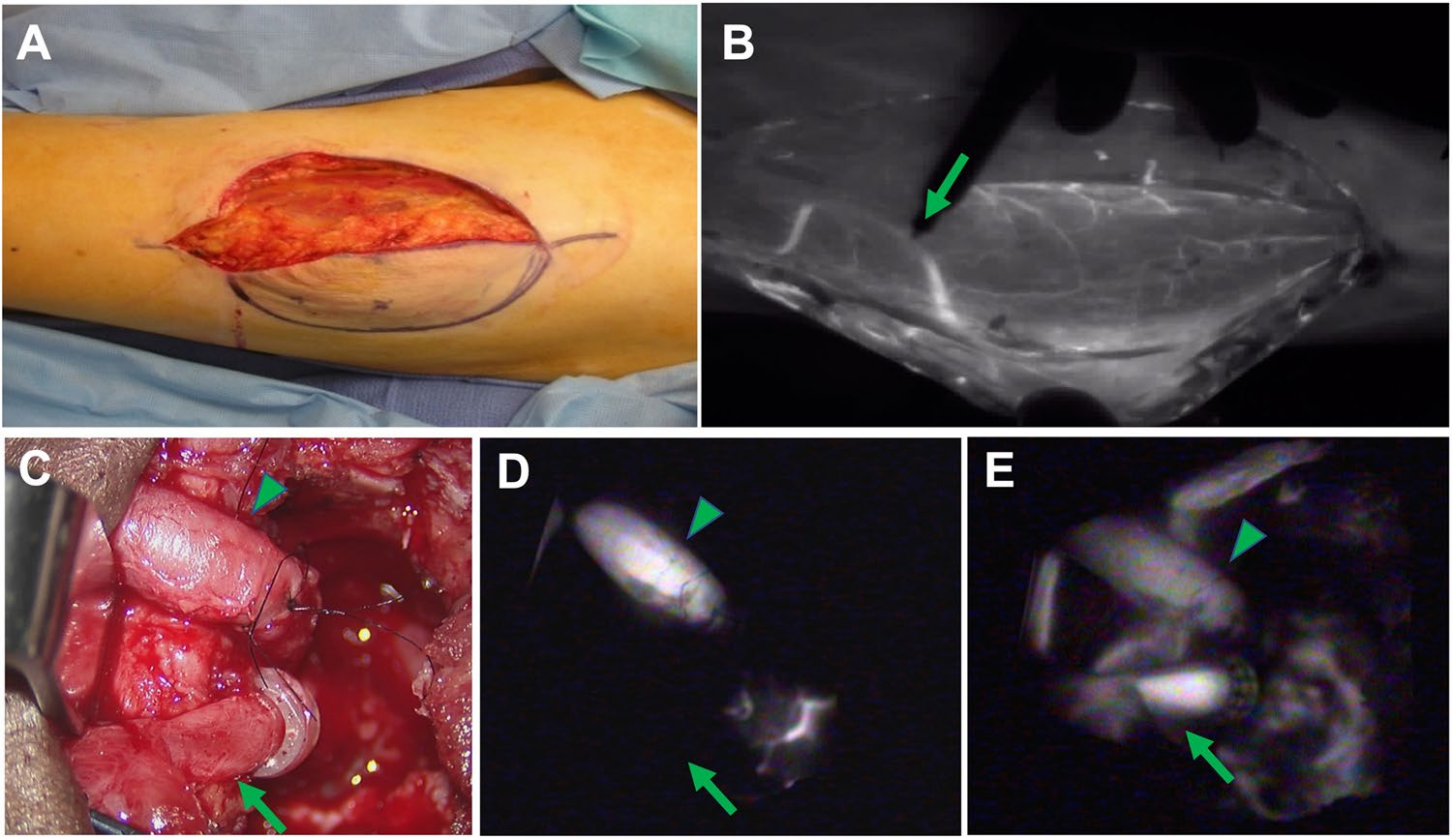
ICG angiography in microvascular reconstructive surgery. **A** Identification of a perforator is crucial during an anterolateral thigh free flap harvest but can be challenging with reflected light visualization only. **B** The perforator can be readily identified (arrow) through its course in muscle by NIR imaging. ICG angiography is also useful in assessment of flow through a microvascular anastomosis. **C** By reflected light imaging, venous flow (arrow) is difficult to ascertain. **D** By ICG angiography and SPY NIR imaging, the artery is observed to have adequate flow (arrowhead), but the venous flow (arrow) is insufficient (**D**). **E** Restoration of venous flow subsequently confirmed by ICG imaging (arrow).

The earliest applications of fluorescence imaging centered around the understanding of vessel anatomy and flow. Given that ICG preferentially binds to albumin, its distribution is retained within the vasculature. Consequently, background signal is minimal and the fluorescence signal emitted highlights vascular anatomy and the characteristics of individual vessels (i.e., vessel caliber, vessel flow, stenosis, and leakage) with a high degree of fidelity. ICG angiography has shown beneficial in nearly every interventional specialty, for example: cardiac surgery, for the assessment of coronary bypass graft patency [26]; ophthalmology, for the diagnosis of choroidal neovascularization [27]; neurosurgery for aneurysm clipping assessment [28]; and reconstructive surgery, to identify flap perforators and assess flow through microvascular anastomoses [29, 30] (Fig. 2).

### Perfusion

**Fig. 3:**
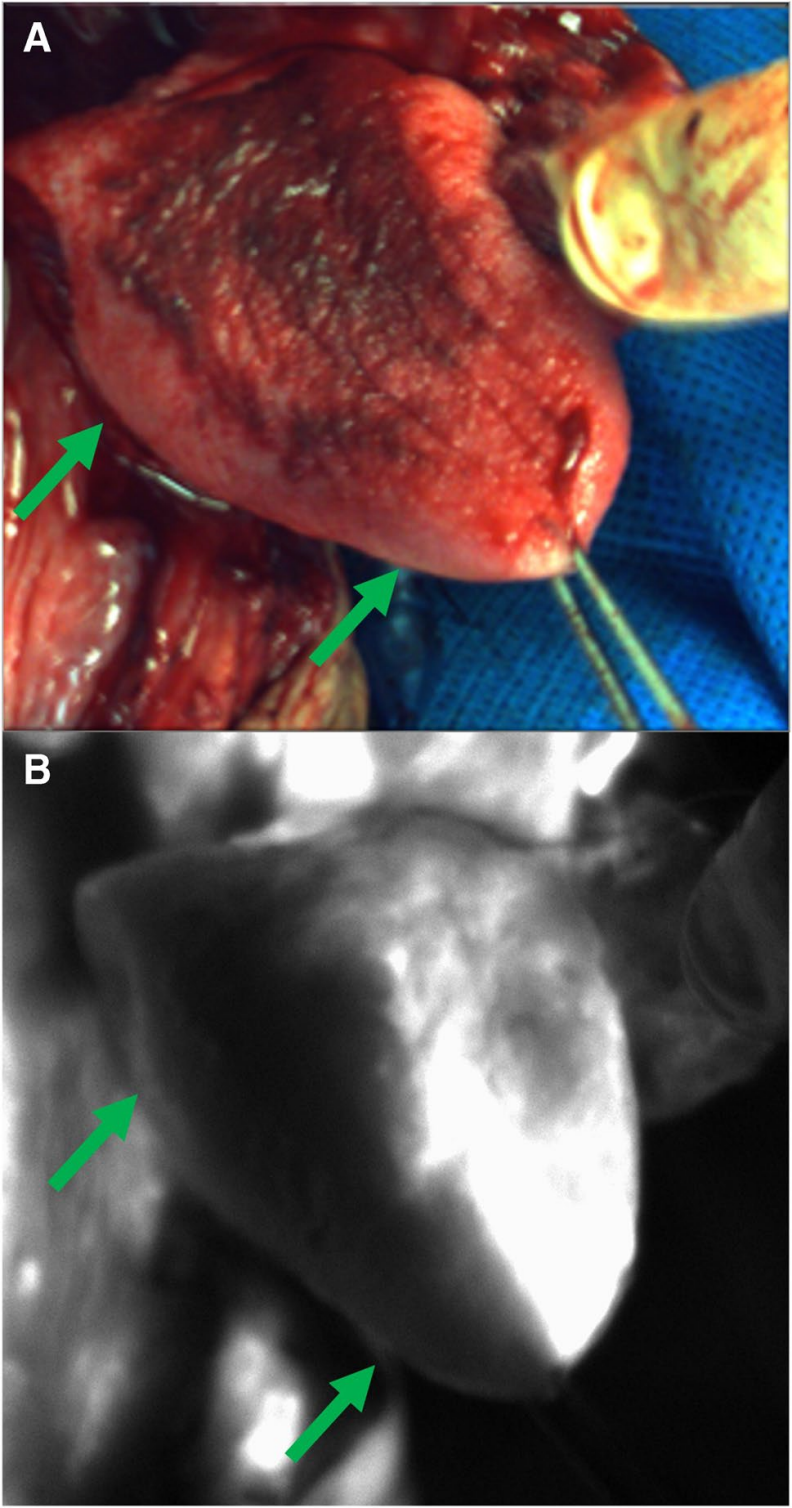
Assessment of tissue perfusion with SPY NIR imaging using intravenous ICG infusion. **A** The right lingual artery was ligated during an oncologic head and neck surgery. Vascularity to the right hemitongue (arrows) appears normal on reflected light images. **B** However, a complete absence of vascular flow is apparent on ICG angiography (**B**).

Tissue perfusion can also be readily assessed using ICG fluorescence. Conventionally, tissue perfusion is assessed using visual markers, such as skin color, capillary refill, and the presence of bright red blood. These markers are not only subjective but are also difficult to assess throughout the entirety of a given tissue. NIR imaging using ICG produces a bright indicator of tissue perfusion, where areas of non-perfusion are readily identified (Fig. 3). This feature has proven especially useful reconstructive surgery, in which the use of ICG has demonstrated up to 88% sensitivity and 97% specificity in predicting free tissue flap necrosis, thereby reducing flap complication rates from 15 to 4% [31].

### Lymphangiography

**Fig. 4:**
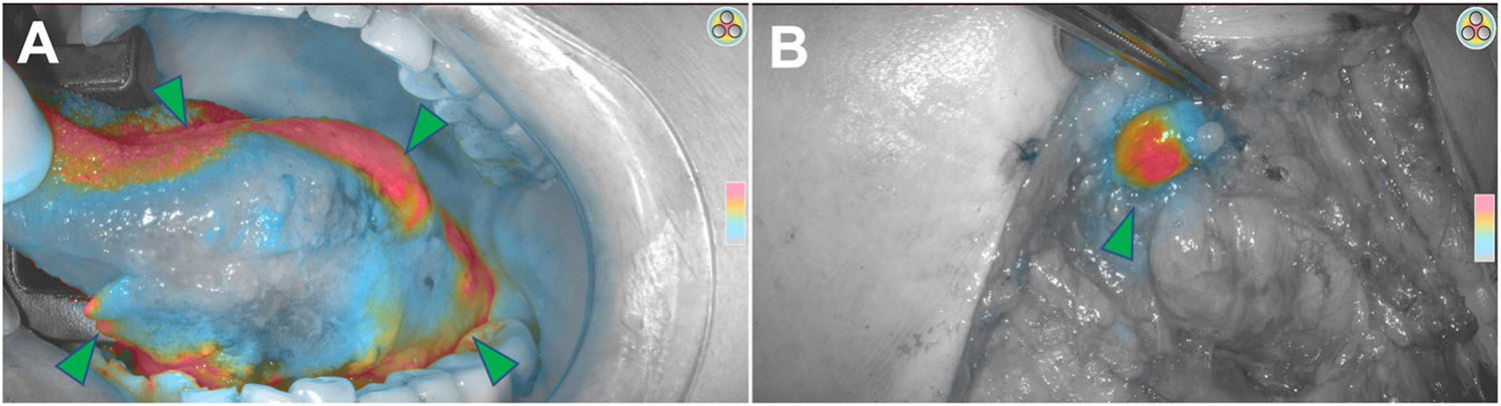
Sentinel lymph node identification using ICG. **A** A total of 5 mg of ICG was injected submucosally around a left tongue squamous cell carcinoma (arrowheads). **B** The neck was subsequently imaged using a handheld SPY NIR imaging system, and a submental lymph node (arrowhead) was identified by fluorescence.

Imaging of the lymphatic system is a unique feature of NIR imaging with ICG. The lymphatic system begins with the lymphatic capillaries of the dermis and other tissues, which coalesce into the larger channels forming the lymphatic system. When injected intradermally, ICG travels the course of these lymphatics and delineates the lymphatic vessels and draining lymph nodes [32]. It thus follows that lymphatic disorders, such as lymphedema, are readily visualized using ICG fluorescence. In addition, ICG has been shown to be a reliable surrogate for methylene blue and standard lymphoscintigraphy with radiotracers in sentinel lymph node mapping [33] (Fig. 4).

### Cholangiography

Due to the hepatic clearance of ICG, NIR visualization of biliary anatomy can be readily achieved. Outlining of the biliary system has shown value in preventing inadvertant injury during common laparoscopic cholecystectomies [34] and robotic resection of gallbladder malignancies [35], and its further utility is being examined in several randomized clinical trials [36–38].

## FGS in Oncologic Surgery

The discovery of fluorescent contrast dyes, development of requisite imaging systems, and experiences gained in wider clinical use have provided the foundation on which FGS in oncologic surgery has developed [39–41]. While angiography, perfusion, lymphagiography, and cholangiography can be performed using non-targeted fluorescent dyes, FGS in oncologic surgery often requires specific targets to adequately differentiate diseased from non-diseased tissue. In the strictest sense, 5-ALA is not a targeted fluorescence agent, but its ability to selectively induce the accumulation of protoporphyrin IX has facilitated its value in oncologic neurosurgery.

**Fig. 5:**
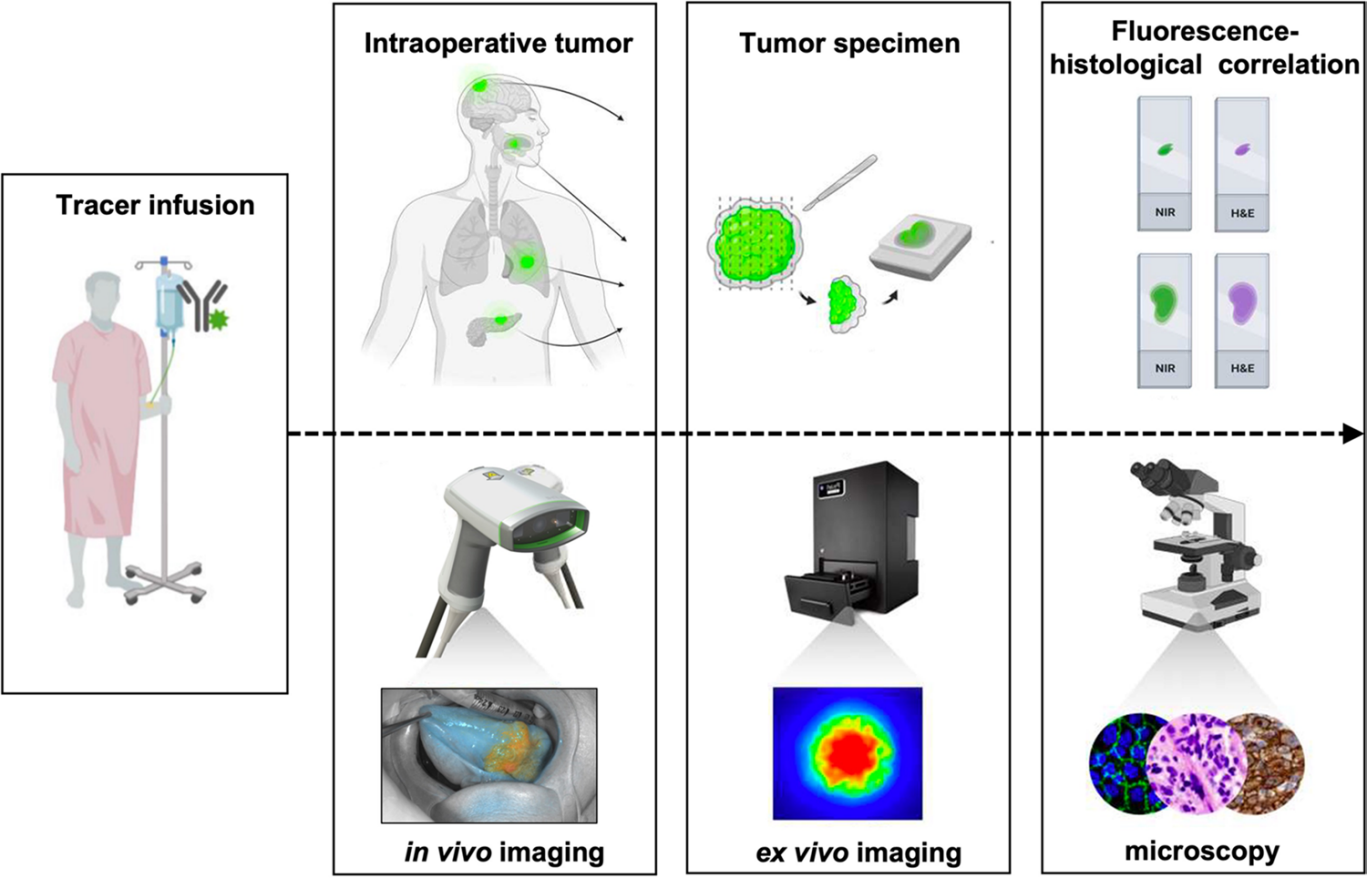
Fluorescence-guided surgery workflow. First, the fluorescence tracer is venously infused preoperatively. During surgery, in vivo open-field camera systems are used to visualize tumor tissue, and to assess adjacent tissue and the wound bed. After the tumor resection, the tumor specimen (i.e., the tumor with a cuff of healthy surrounding tissue) is imaged within a closed-field ex vivo fashion. Thereafter, during histological assessment, the fluorescence is traced within the tumor specimen to ascertain if fluorescence areas correspond with tumor areas.

The potential value of a tumor-specific optical guide rests in its ability to affect surgical decision-making. Given that oncologic surgery is largely guided by visual and tactile cues, it follows that an additional layer of tumor-specific visualization could help navigate tumor margin resection, diagnosis of secondary tumors, and regional lymph node disease. The incorporation of fluorescence-guidance in the surgical workflow ideally does not disrupt standard practice, but rather augments the ability of the surgeon to plan tumor resection, assess the resection bed, and analyze the resected specimen margins. After administration of a fluorescent agent hours to days before surgery, tumor tissue can be visualized throughout the operation using specialized NIR camera systems. Figure 5 describes the imaging workflow through the different stages of the procedure distinguishing in vivo imaging (pre-incision, during resection, and wound bed inspection) and ex vivo imaging (back-table or pathological specimen imaging).

In vivo fluorescence imaging has demonstrated promise to improve surgical decision-making by enhancing the visualization of tumor margins. Stummer et al. have shown that oral administration of 5-ALA helped to delineate the periphery of high-grade gliomas intraoperatively, leading to improved tumor margin clearance and higher 6-month progression free survival, as compared to surgery using merely white light (41.0% vs 21.1%) [17]. Besides identification of primary tumor margins, fluorescence imaging could also be employed in the detection of satellite lesions, local metastases (i.e., lymph nodes), or secondary tumors. Patients with head and neck squamous cell carcinoma (HNSCC) often develop secondary tumors as the greater portion of the oral mucosa is exposed to the carcinogenic effects of tobacco and alcohol. Work performed by our group illustrated results recapitulating that of Stummer et al., in which in vivo use of targeted fluorescence imaging during HNSCC resection led to improved surgical decision-making in three of 14 cases (21.4%) [42]. Fluorescence imaging identified a close tumor margin in one case, and in two other cases, helped to identify unanticipated extent of disease. Although patient numbers are relatively small, this study underlines how intraoperative decision-making can be improved by in vivo fluorescence imaging.

Recently, the FDA approved another intraoperative imaging guide, pafolacianine (OTL38, Cytalux; Table 1), a fluorescent agent which enhances the intraoperative localization of difficult-to-identify ovarian cancer tissue [8, 43]. After intravenous administration, the agent binds to the folate receptor which is differentially expressed on ovarian cancer cells, improving the surgeon’s ability to identify cancer lesions using an open-field NIR camera system. In a randomized, multi-center, open-label study, 134 women, with or with high clinical suspicion, of ovarian cancer, underwent surgery with intraoperative fluorescence imaging of pre-administered pafolacianine. In 26.9% of these cases, at least one cancerous lesion was detected which had gone unnoticed using merely visual and tactile inspection [8].

Although in vivo imaging provides the user with beneficial information concerning primary location, tumor margins, and unanticipated extent of disease, it typically provides qualitative data. This is due to a variety of uncontrolled, environmental factors which interrupt the fluorescence signal, such as ambient light from operating room overhead lights, camera-to-target distance, and camera angle. Quantitative imaging data, on the other hand, enables signal measurements and allows for inter-patient comparison. Ex vivo imaging is a method that permits such quantitative imaging within a controlled closed-field environment. Such ex vivo imaging systems have fixed camera distances and does not utilize ambient light, which minimizes reflectance and results in reproducible quantitative data.

Note that in vivo and ex vivo imaging methods can and should act as complementary imaging modalities. After the tumor is resected using in vivo fluorescence imaging guidance, the resected tumor specimen (i.e., tumor with a cuff of healthy adjacent tissue surrounding the tumor) is imaged ex vivo within a closed-field device on the back-table in the operating room or at the pathology department. During resection of solid tumors, defining sufficient tumor margins remains difficult due to the mostly small field of view (i.e., oral cavity, laparoscopy, or robotic approaches) and proximity of critical structures at risk of iatrogenic injury. In vivo and ex vivo together provide a fluorescence surface map of the tumor, wound bed, and tumor specimen and enable visualization of potentially tumor harboring margins in need of further clinical assessment [42].

## Opportunities for Further Development

While the advantages of FGS are clear, the limitations of its use in oncologic surgery should be addressed. Cancer is a largely heterogeneous entity with a variety of gene expression profiles between two tumors of the same cancer type as well as among different cancer types. To achieve high sensitivity, a homogenous distribution of fluorescence signal deriving from target cells is required. We can see that in many of the FDA-approved agents, the tracer is directed towards a ligand that is present on the majority of cancer cells (Table 1), generating a consistent fluorescent signal. Furthermore, in cancer types with numerous gene expression variations indicating highly heterogenic tumors, a multi-plexed approach to tumor characterization could increase signal by deploying multiple labeled antibodies [44].

In addition to improving the tumor targeting ability of fluorescent agents, continued development of NIR imaging devices is also necessary. As many agents have proven to be highly specific for various cancers, sensitivity is for a great part defined by the imaging system’s ability to register the signal coming from targeted tissue. Consequently, we have seen a tremendous upsurge in imaging system performances over the past 5 years [45]. Besides imaging system capabilities, sensitivity can be increased by reducing background signal coming from adjacent non-targeted tissues. Furthermore, improvement in the strategies to display fluorescence images should be considered. Current intraoperative imaging systems display fluorescence images on a separate display monitor, drawing the surgeon’s vision away from the surgical bed. Integrated visual systems, or “mixed-reality” devices such as the Microsoft Hololens, can be utilized to project fluorescence data over the surgeon’s natural vision for seamless real-time fluorescence navigation. Such devices can further augment the surgical field with presurgical imaging, such as positron emission tomography (PET), computed tomography (CT), and single-photon emission computed tomography (SPECT) [46].

**Fig. 6:**
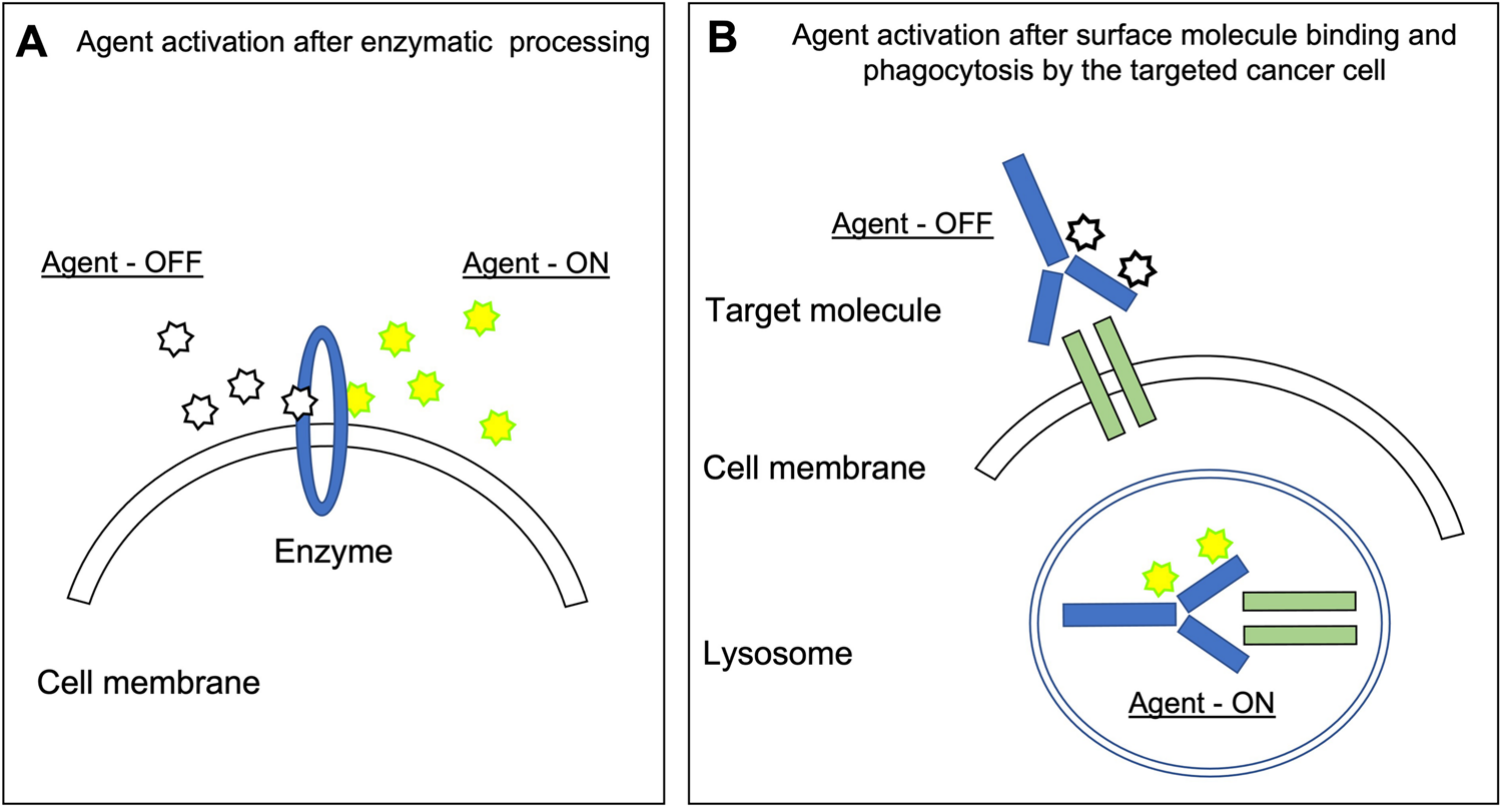
Activatable fluorescence probes. **A** Enzyme-activable probes allow for fluorescence signal to emit only in the presence of specific enzymatic activity. **B** An alternative strategy allows for fluorescence activation after incorporation of the agent by surface molecule binding and lysosome phagocytosis by the targeted cancer cell.

The fundamental drawback of agents that emit signal regardless of their proximity to the target (so-called always-on agents) is that background signal is fairly high. To improve signal deriving from the targeted tissue, it is essential to maximize signal from targeted tissue and minimize that of surrounding adjacent tissue to increase the signal-to-background ratio. Technologies such as short-wave infrared imaging (SWIR) [47, 48] and lifetime fluorescence imaging [49] are intriguing methods to potentially improve signal-to-background ratios. In addition, “activatable” or so-called smart-probes holds promise to improve tumor specificity and decrease background signal. These probes capitalize on physiological differences between cancerous and normal tissue, or utilize fluorescence quenching (Fig. 6). In the latter, activatable agents, such as γ-glutamyl transferase (GGT), are in a quenched state until arrival at the targeted tissue whereas cleavage by cell surface enzymes enables signal emission (Fig. 6A) [50]. The main disadvantage inherent to fluorophore activation by cell membrane enzymes is that the fluorophore remains extracellular and detached from the cell membrane, and as diffusion takes place in all tissues, these smart-probes lack capabilities in tumor margin detection. Figure 6B illustrates an alternative strategy in which endocytosis of the quenched agent occurs. After endocytosis, the low pH during endolysosomal processing within the lysosome ensures the release of the fluorophore from its quenched state enabling selective signal transmission from within targeted tumor cells [51, 52].

**Fig. 7:**
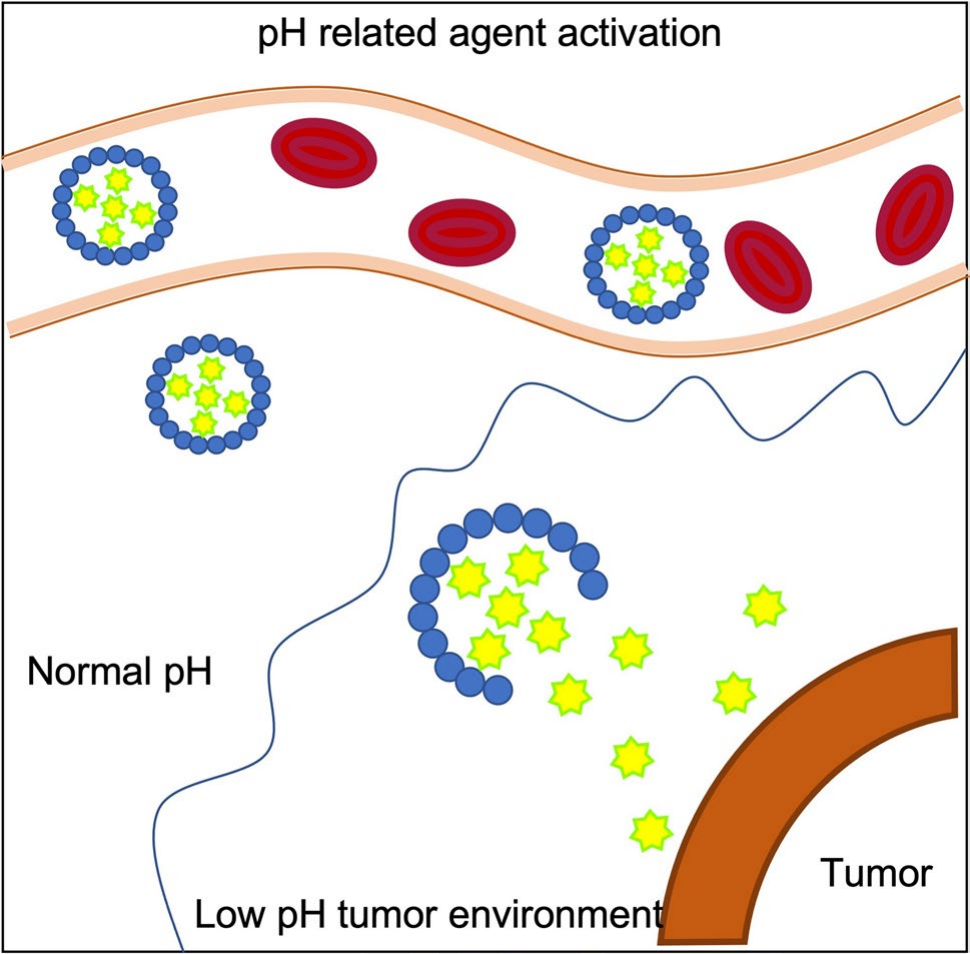
pH induced fluorescence agent activation. The agent is in quenched state within a micelle in a pH neutral environment. As the micelle reaches the acidic tumor environment, it dissolves and the agent starts to emit its photons when excitation takes place.

However sensitive these techniques are, they often lack broad tumor application, as most of fluorescence-guided imaging evolves around fluorophore labeled small molecules, nanobodies, peptides, or nanoparticles against cell surface receptors on specific cancer cells [53, 54]. While targeting the features of specific tumor types (i.e., ovarian cancer and high-grade glioma) has its advantages, a more encompassing approach would be to exploit the dysregulated environment that solid tumors demonstrate in common. Due to intrinsic tumor factors such as hypoxia caused by anaerobic glycolysis and angiogenesis, the tumor environment gives rise to a significantly lower pH compared to nearby healthy tissue. For example, Voskuil et al. utilized pH-sensitive amphiphilic polymers that generate a fluorescent signal in response to a low pH [55]. If the smart-probe resides in a normal pH environment, the polymers self-assemble in micelle formation (Fig. 7). Within the micellar core, the fluorophores are tucked together in a quenched state. When the nanometer-sized micelles enter the tumor acidic environment, they irreversibly change shape and the fluorophores shift from their quenched form.

Furthermore, as the field of FGS quickly progresses, various bridges can be made between surgical oncology using fluorescent agents and conventional nuclear imaging using radionuclides. By combining both a fluorescence dye and a radionuclide to targeting agent, a single tracer could be exploited for presurgical nuclear imaging as well as intraoperative fluorescence imaging [56]. The first in-human example of such dual-modality agent is ^68^ Ga-NOTA-BBN-IR-800CW which targets gastrin-releasing peptide receptors in glioblastomas [57]. After Li and colleagues illustrated the feasibility of this fruitful collaboration between nuclear medicine and FGS, multiple dual-modality agents have subsequently undergone development.

## Summary

The past decade has seen tremendous research into the development of new fluorescence technologies which have the potential to revolutionize surgical practice. Reinforced by advances in molecular imaging technology, the growing experience of phase I/II trials and recent FDA approvals of optical guides for oncologic surgery, there is a need for a clear pathway to ensure successful clinical translation of new technology. Elements of this pathway include, but is not limited to: (a) approval of drug and devices through the FDA; (b) approval of reimbursement protocols by the Centers for Medicare & Medicaid Services (CMS); (c) research sponsorship from the National Cancer Institute (NCI); and (d) engagement and support of medical and imaging societies, academic institutions, and industry.

While navigating the regulatory and approval processes can be challenging, collaboration among the aforementioned groups have sought to provide clarity and insight into these processes [58]. In particular, it is important that we acknowledge the efforts of the late Sanjiv S. Gambhir and many others who have strived to initiate inclusive discussion among stakeholders, increase awareness of regulatory requirements, and advocate for streamlined approval processes.
